# An open-label randomized controlled trial evaluating the efficacy of chloroquine/hydroxychloroquine in severe COVID-19 patients

**DOI:** 10.1038/s41598-021-88509-9

**Published:** 2021-04-27

**Authors:** Álvaro Réa-Neto, Rafaella Stradiotto Bernardelli, Bruna Martins Dzivielevski Câmara, Fernanda Baeumle Reese, Marcos Vinicius Oliveira Queiroga, Mirella Cristine Oliveira

**Affiliations:** 1 Federal University of Paraná (UFPR); CEPETI, Center for Study and Research in Intensive Care Medicine, Rua Monte Castelo, 366, Curitiba, CEP 82590-300 Brazil; 2 CEPETI, Center for Study and Research in Intensive Care Medicine, Rua Monte Castelo, 366, Curitiba, CEP: 82590-300 Brazil

**Keywords:** Infectious diseases, Respiratory distress syndrome

## Abstract

Despite several studies designed to evaluate the efficacy of chloroquine and hydroxychloroquine in the treatment of coronavirus disease 2019 (COVID-19), there is still doubt about the effects of these drugs, especially in patients with severe forms of the disease. This randomized, open-label, controlled, phase III trial assessed the efficacy of chloroquine or hydroxychloroquine for five days in combination with standard care compared to standard care alone in patients hospitalized with severe COVID-19. Chloroquine 450 mg BID on day 1 and 450 mg once daily from days 2 to 5 or hydroxychloroquine 400 mg BID on day 1 and 400 mg once daily from days 2 to 5 were administered in the intervention group. Patients were enrolled from April 16 to August 06, 2020, in 6 hospitals in southern Brazil. The primary outcome was the clinical status measured on day 14 after randomization with a 9-point ordinal scale. The main secondary outcomes were all-cause mortality; invasive mechanical ventilation use; the incidence of acute renal dysfunction in 28 days; and the clinical status of patients on days 5, 7, 10 and 28. All patients with a positive RT-PCR result for severe acute respiratory syndrome coronavirus 2 (SARS-CoV-2) were analyzed (modified intention to treat (mITT) population). Arrythmias and cardiovascular complications were assessed as safety outcomes. A total of 105 patients were enrolled and followed for 28 days. The trial was stopped before reaching the planned sample size due to harmful effects. Patients in the intervention group had a worse clinical outcome on the 14th day (odds ratio (OR) 2.45 [1.17 to 4.93], p = 0.016) and on the 28th day (OR 2.47 [1.15 to 5.30], p = 0.020). Moreover, the intervention group had higher incidences of invasive mechanical ventilation use (risk ratio (RR) 2.15 [1.05 to 4.40], p = 0.030) and severe renal dysfunction (KDIGO stage 3) (RR 2.24 [1.01 to 4.99], p = 0.042) until the 28th day of follow-up. No significant arrythmia was noted. In patients with severe COVID-19, the use of chloroquine/hydroxychloroquine added to standard treatment resulted in a significant worsening of clinical status, an increased risk of renal dysfunction and an increased need for invasive mechanical ventilation.

**Trial Registration:** ClinicalTrials.gov, NCT04420247. Registered 09 June 2020—Retrospectively registered, https://www.clinicaltrials.gov/ct2/show/study/NCT04420247.

## Introduction

The novel coronavirus, severe acute respiratory syndrome coronavirus 2 (SARS-CoV-2), causing coronavirus disease 2019 (COVID-19), has already infected tens of millions of people around the world and killed more than a million people (approximately 3%)^1^. Patients with risk factors or severe forms of COVID-19 have more than a 30% chance of dying. Many antiviral or anti-inflammatory drugs have been studied to find a way to control the poor outcomes of COVID-19^2^. Some drugs have demonstrated in vitro activity against SARS-CoV-2 and potential clinical benefits in small and/or observational studies^3^. In this scenario, experiments with chloroquine (Clq) and its derivative hydroxychloroquine (HClq) revealed that these drugs are able to decrease viral replication and are associated with faster SARS-CoV-2 elimination^4^.

Clq is a 9-aminoquinoline known since 1934. Apart from its recognized antimalarial effects, the drug has some biochemical properties that, in theory, are applicable against some viral infections. Clq exerts direct antiviral effects, inhibits pH-dependent steps of the replication of several viruses and has immunomodulatory effects, suppressing the production/release of tumor necrosis factor and interleukin 6, which mediate the inflammatory complications of these viral diseases. In addition, Clq has a well-studied toxicity profile^2,5^.

Previous randomized and controlled clinical trials have not proven benefits of Clq/HClq for postexposure prophylaxis or the treatment of COVID-19 in mild or moderate cases^6–8^. The RECOVERY trial, in which 75% of the sample comprised patients with severe COVID-19, showed no difference in mortality, although patients who received HClq were more likely to need invasive mechanical ventilation (IMV)^9^.

Thus, an open-label randomized controlled trial (RCT) was conducted with only critically ill patients hospitalized with severe COVID-19^10^ to evaluate the efficacy of five-day use of Clq or HClq in combination with standard care^11^ compared to standard care alone in improving the clinical status on the 14th day, which was evaluated by a 9-point ordinal scale recommended by the World Health Organization (WHO)^12^.

## Methods

### Study design and overview

A multicenter, open-label, RCT was conducted in six hospitals in Curitiba, Brazil, by the Center of Study and Research on Intensive Care Medicine (CEPETI). The trial was approved by the Brazilian National Commission for Ethics in Research (3960331). All methods were carried out in accordance with relevant guidelines and regulations. All participants, or their legal representatives, provided written informed consent. This trial is registered at ClinicalTrials.gov (NCT04420247). The trial protocol is in the related files section.

After the initiation of randomization, the protocol was amended once to authorize informed consent through a phone call by the patient´s legal representative when applicable. The change was authorized by the regulatory ethical agencies of Brazil.

### Patients

Patients admitted to the intensive care unit (ICU) or acute care rooms in 6 hospitals were consecutively evaluated. Those aged 18 years or more were eligible if they had flu symptoms (runny nose, dry or productive cough, sore throat and/or fever) associated with one of the following manifestations: clinical need for supplemental oxygen for dyspnea, pulse oxygen saturation ≤ 94% on room air, pulmonary computed tomography findings compatible with COVID-19^13^ or the necessity of mechanical ventilation (MV) and a diagnosis of SARS-CoV-2 infection confirmed by molecular analysis or RT-PCR performed at admission.

Patients with a significant history of cardiopathy or any kind of arrhythmia, psoriasis, seizure, G6PD deficiency, myasthenia gravis, ALT/AST > 5 times the normal values, creatinine clearance < 30 ml/min/1.73 m^2^, pregnancy or lactation or known Clq/HClq allergy were excluded. An electrocardiogram (ECG) was performed on all patients at admission, and those with any arrhythmia or an abnormally prolonged QT interval were excluded. Patients with a clinical history of arrhythmia were also excluded, even if they did not present ECG changes at baseline.

### Trial procedures

Patients were randomized (1:1) within 48 h of admission to take Clq or HClq for 5 days plus standard treatment or control (standard treatment only).

Randomization was performed in blocks of variable size (2, 4 and 6) in a centralized web-based automated system and stratified by site and whether the patient was on IMV. The allocated group was disclosed to the investigator only after all information regarding patient enrollment had been recorded in the web system. Clq is available to patients in public hospitals in Brazil. Patients in private centers, however, received HClq because Clq was not available to them. The drugs were administered by mouth or gastric/enteral tube at the following dosage: Clq 450 mg BID on day 1 and 450 mg once daily from day 2 to 5 and HClq 400 mg BID on day 1 and 400 mg once daily from day 2 to 5. These dosages of Clq/HClq were chosen because they had a lower incidence of adverse effects, were proposed in several international protocols and were recommended by the Brazilian Ministry of Health by the time the trial began^14–17^. In cases where the patient was discharged in less than 5 days, the medication was provided to them, accompanied by a form to fill in with the doses to be taken on the following days and guidelines.

Each study site was encouraged to follow the best practice guidelines for the care of critically ill patients with COVID-19^11^. The use of glucocorticoids, antibiotics and antiviral agents was allowed. All clinical interventions, such as ventilatory support, laboratory testing, and hemodynamic management, were left at the discretion of the ICU team for both groups.

For safety reasons, attending physicians were instructed to observe changes in cardiac monitoring and, upon the suspicion of any abnormality, to perform an ECG immediately. In addition, a routine ECG was requested on the 6th day after treatment initiation for all hospitalized patients.

### Clinical and laboratory data

Data were collected daily, from randomization to day 28, in an electronic case-report form system. Data on demographic characteristics, physiological variables, medication use before randomization, the timing of symptoms and other clinical and laboratory information were collected, including the results of SARS-CoV-2 tests. For patients who had been discharged earlier, a telephone call was made on days 5, 7, 10, 14 and 28. During the telephone visits, patients were asked about the presence of symptoms and answered the 12-item version of the WHO Disability Assessment Schedule (WHODAS 2.0)^18,19^.

### Outcomes

The primary outcome, as suggested by WHO’s Master Protocol^12^, was the clinical status of patients measured on day 14 with a 9-point ordinal scale as follows: (0) nonhospitalized and no clinical or virological evidence of infection; (1) nonhospitalized and no limitation on activities; (2) nonhospitalized, but with limitation on activities; (3) hospitalized, but not requiring supplemental oxygen; (4) hospitalized and on oxygen via mask or nasal prongs; (5) hospitalized, on noninvasive ventilation or high-flow oxygen or pressure support ventilation in weaning mode; (6) hospitalized, intubated and on MV; (7) hospitalized on MV and additional organ support (renal replacement therapy, vasoactive drugs or extracorporeal membrane oxygenation), and (8) dead.

Secondary outcomes were all-cause 28-day mortality; the clinical status of patients on days 5, 7, 10 and 28 according to the 9-point ordinal scale; the length of ICU stay; the length of hospital stay; the number of ventilator-free days until day 28 or death; the incidence of acute renal dysfunction (i.e., Kidney Disease: Improving Global Outcomes (KDIGO) stage 3); the incidence of coagulopathy (platelet count < 150.0 × 10^9^/L and/or international normalized ratio (INR) > 1.5 and/or kaolin partial thromboplastin time (KPTT) > 35 s); the incidence of intubation and IMV anytime until the 28th day; and the Sequential Organ Failure Assessment (SOFA) score, C-reactive protein (CRP) level and neutrophil/lymphocyte ratio at admission and on days 5, 7, 10, 14 and 28 during hospitalization.

The safety outcome of interest was arrhythmia identified by the attending physician at the time of occurrence, confirmed by ECG. Any kind of arrhythmia was taken into account.

### Sample-size calculation and protocol changes

The study started with provisional initial sampling in compliance with the WHO Master Protocol. According to the following distribution probabilities in the 9-point ordinal scale (16.1% for 0, 13.7% for 1; 33.2% for 2, 19.6% for 3, 7.1% for 4, 3.1% for 5, 2.3% for 6, 2.3% for 7 and 2.25% for 8), a sample of 394 patients was estimated for an effect size with an odds ratio (OR) of 2, considering a two-tailed 5% significance level and the statistical power of 85% and allowing a 10% sample loss^12^. As predicted in the study protocol, the sample size was recalculated based on the primary outcome results obtained in the interim analysis of the first 105 patients positive for SARS-CoV-2 infection. In this scenario, an OR less than 1.0 represents a clinical improvement assessed by the ordinal scale in the intervention group compared to the control group.

Although we intended to randomize 394 patients at the beginning of the trial, in our interim analysis with 105 patients, we found a statistically significant difference for the primary outcome and for two secondary outcomes (a higher proportion of patients with renal dysfunction (KDIGO stage 3) and higher progression to a need for IMV) in patients in the intervention group. Therefore, the steering committee decided to discontinue recruitment for safety reasons. Then, the interim analysis became the definitive analysis.

As preplanned in the study protocol (see trial protocol in the related files section), all statistical methodologies provided for the final analysis were performed in the interim analysis and are described below.

### Statistical analysis

For the analysis of the primary outcome, the effect of the intervention on the 9-point ordinal scale values was presented as an OR and confidence interval (CI) derived from an ordinal logistic regression, assuming proportional ORs adjusted for age and severity at baseline (under MV or not at randomization) on day 14. The same regression model was used to analyze the secondary outcomes according to the 9-point ordinal scale on days 5, 7, 10 and 28 after randomization.

The effect of the intervention on mortality on day 28 and the incidences of MV, acute renal dysfunction (i.e., KDIGO stage 3) and coagulopathy at any moment until the 28th day are reported as proportions, and differences between groups are reported as risk ratios (RRs) with CIs, calculated by using the Wald likelihood test. Kaplan–Meier survival curves were constructed to show the cumulative incidence of IMV, acute renal dysfunction, and mortality over the 28-day period. The effect of the intervention on the number of MV-free days and the length of stay in the ICU and hospital for 28 days was compared by median differences calculated with a quantile regression based on an asymmetric Laplace distribution, with p values from the Wilcoxon rank-sum test. The same analysis was used to compare SOFA scores, CRP values and neutrophil/lymphocyte ratios between groups during hospitalization on days 5, 7, 10, 14 and 28 after randomization. A two-tailed *p* value less than 0.05 was considered statistically significant in all analyses.

Additional statistical analyses were performed to describe and compare the outcomes among the Clq, HClq and control groups, since the intervention group was composed of patients who received Clq or HClq. The details are described in the Additional Statistical Analysis section of the supplementary material. There were no missing values for the primary outcome; therefore, imputation was not necessary. The analysis was performed using Stata software, version 17.

## Results

**Figure 1:**
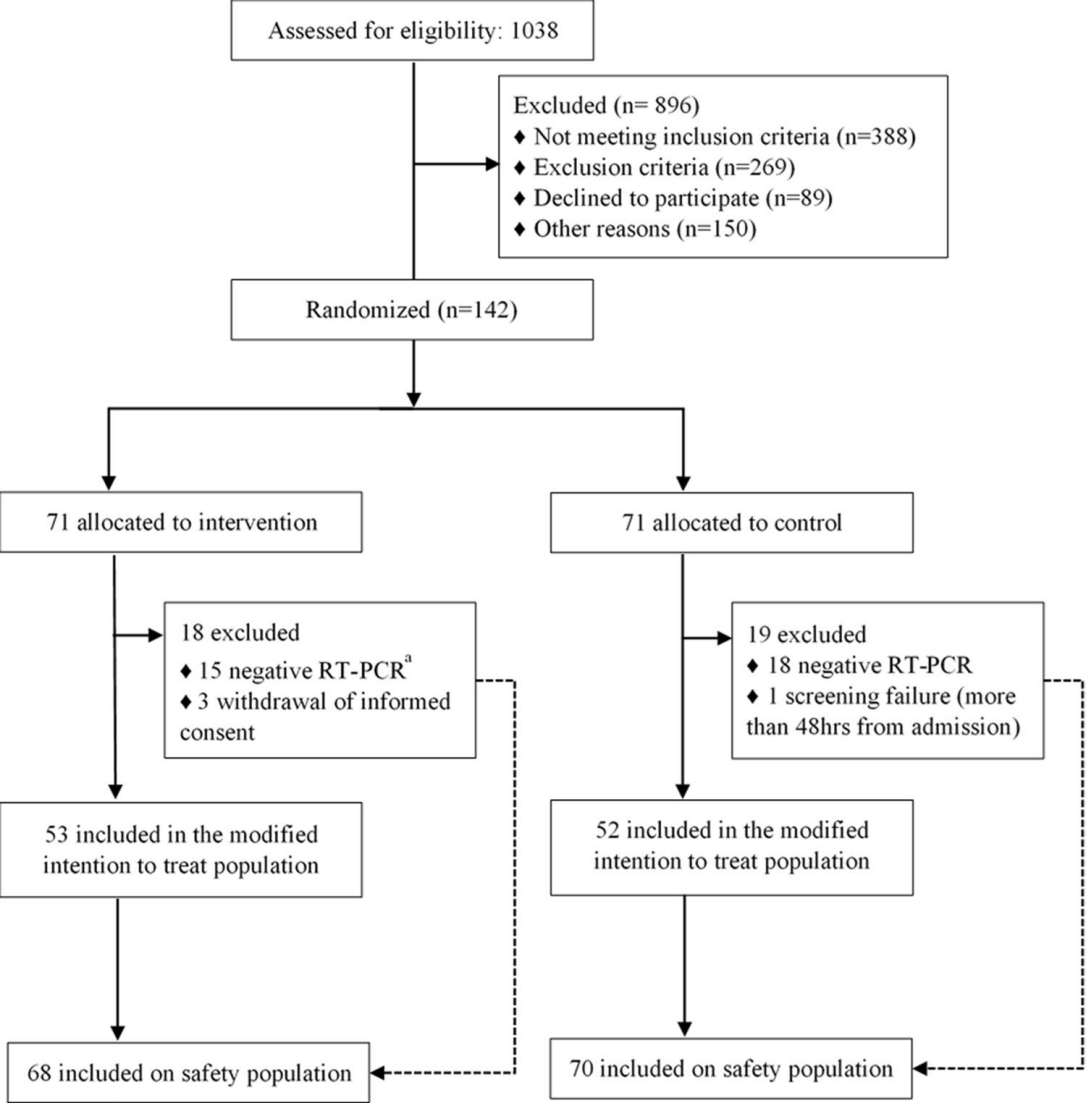
Trial profile. ^a^One of these patients did not receive at least two doses of the assigned treatment and was not included in the safety analyses.

From April 16 to August 06, 2020, we screened 1038 patients consecutively. Of these, 896 were excluded, and 142 were enrolled in the trial, of whom 84.5% were admitted to the ICU at the time of randomization. Later, 3 patients were excluded because of the withdrawal of informed consent, and one was excluded due to screening failure. Only patients with suspected severe COVID-19 were randomized (intention-to-treat (ITT) population), but efficacy analysis was performed exclusively on patients with SARS-CoV-2 infection confirmed through molecular or serological testing (modified intention-to-treat (mITT) population). Acute SARS-CoV-2 infection was confirmed in 105 patients (53 in the Clq/HClq group and 52 in the control group). All of these patients completed the 5-day allocated treatment unless premature death occurred. Only one patient in the control group was lost to follow-up on day 28. The safety analysis included all randomized patients (ITT population, n = 138), even those who tested negative for SARS-CoV-2 infection (n = 33) but received the intervention (Clq/HClq) until the test result was returned (Fig. 1).

Baseline characteristics were well balanced between the two groups (Table 1). The median age of the patients was 53 years, 66.7% of all the included patients were men, and the median time from symptom onset to randomization was 7 days. A total of 81.9% of the patients were receiving supplemental oxygen at baseline, and 18.1% were mechanically ventilated. Baseline characteristics were also balanced among the Clq, HClq and control groups (Supplementary Table S1).

**Table 1:** Baseline characteristics and concomitant treatments during hospitalization in the modified intention to treat population.

Characteristics	Clq/HClq group (n = 53)	Control group (n = 52)
Age, mean (SD), years	54.7 (12.1)	52.8 (12.6)
Sex male, no. (%)	36 (67.9)	34 (65.4)
Body mass index, mean (SD)	31.3 (5.7)	30.8 (5.5)
**Comorbidities, no. (%)**^**a**^		
Hypertension	19 (35.8)	21 (40.4)
Diabetes mellitus	11 (20.8)	16 (30.8)
Chronic lung disease^b^	6 (11.3)	3 (5.8)
Immunocompromised state^c^	2 (3.8)	4 (7,6)
Charlson score, median (IQR)	0.5 (0–1)	0.7 (0–1)
SpO_2_ < 94%, no. (%)	51 (96.2)	49 (94.2)
IMV at baseline, no. (%)	9 (17)	10 (19.2)
In ICU at baseline, no. (%)	44 (83)	41 (78.8)
Pulmonary ground-glass opacities in CT scan, no. (%)	52 (98.1)	49 (94.2)
Time from symptom onset to randomization, median (IQR), days	8 (5.5–10)	7 (5–10)
APACHE II score, median (IQR)	9 (5.5–12)	8 (5–12.8)
SOFA score, median (IQR)	3.0 (2–4)	2.5 (1–4)
KDIGO stage, no. (%)		
0	44 (83)	40 (76.9)
1	7 (13.2)	9 (17.3)
2	1 (1.9)	2 (3.8)
3	1 (1.9)	1 (1.9)
Coagulopathy at baseline, no. (%)	8 (15.1)	9 (17.3)
**Score on nine-point ordinal scale, no. (%)**^**d**^		
3: hospitalized, but not requiring supplemental oxygen	1 (1.9)	0 (0)
4: hospitalized and on oxygen by mask or nasal prongs	43 (81.1)	42 (80.8)
5: hospitalized, on NIPPV, HFNC or support pressure MV in weaning mode	0 (0)	0 (0)
6: hospitalized, intubated and on MV	1 (1.9)	3 (5.8)
7: hospitalized on MV and additional organ support (hemodialysis and/or vasoactive drugs and/or ECMO)	8 (15.1)	7 (13.5)
**Laboratory variables**		
Hemoglobin, mean (SD), g/dL^e^	13.5 (1.6)	13.9 (1.3)
White blood cell count, median (IQR), × 10^9^/L^e^	7.8 (6.1—10.8)	7.4 (5.2—9)
Lymphocyte count, median (IQR), × 10^9^/L^e^	0.97 (0.66—1.40)	0.87 (0.56—0.90)
Neutrophil/lymphocyte ratio, median (IQR)^e^	6 (3.9—10.1)	6.5 (4.0—9.7)
Platelet count, mean (SD), × 10^9^/L^e^	222.0 (79.5)	217.2 (78.0)
Serum creatinine, median (IQR), mg/dL	0.78 (0.65—1.02)	0.78 (0.63—1.00)
CRP, median (IQR), mg/L^f^	101.8 (65.0—158.0)	84 (41.5—151.0)
D-dimer, median (IQR), nmol/L^g^	893.8 (432—1645.8)	821 (409 -1601.3)
**Concomitant medications**		
Corticosteroids, no. (%)	37 (69.8)	39 (75)
Oseltamivir, no. (%)	27 (50.9)	27 (51.9)
Azithromycin, no. (%)	51 (96.2)	43 (82.7)

*SpO*_*2*_ pulse oxygen saturation; *ICU* intensive care unit; *MV* mechanical ventilation; *IMV* invasive ventilation; *CT* computed tomography; *APACHE II* Acute Physiology and Chronic Health disease Classification System II; *SOFA* Sequential Organ Failure Assessment; *KDIGO* Kidney Disease: Improving Global Outcomes; *HFNC* high-flow nasal cannula; *NIPPV* noninvasive positive-pressure ventilation; *ECMO* extracorporeal membrane oxygenation; *CRP* C-reactive protein, *SD* standard deviation; *IQR* interquartile range; *Clq* chloroquine; *HClq* hydroxychloroquine.

^a^No participants had any of the following comorbidities: chronic renal failure; peripheral vascular insufficiency; or heart, liver, rheumatic, or hematological disease.

^b^Considering asthma or chronic obstructive pulmonary disease.

^c^Considering cancer or human immunodeficiency virus infection.

^d^Only hospitalized patients were eligible for the trial; therefore, patients who had scores of 0, 1, 2 or 8 on a nine-point ordinal scale were not eligible.

^e^One missing data point in the Clq/HClq group.

^f^Three missing data points in the Clq/HClq group and one missing data point in the control group.

^g^27 missing data points in the Clq/HClq group and 25 in the control group.

At randomization, the proportions of patients receiving azithromycin, corticosteroids or antiviral agents (oseltamivir) were similar in both groups (Table 1). Other therapeutic strategies, such as remdesivir, tocilizumab and convalescent plasma, were not available for any participant in the study. From July 17th on, after the publication of the RECOVERY study^20^, all patients were taking dexamethasone (intravenously or orally).

**Figure 2:**
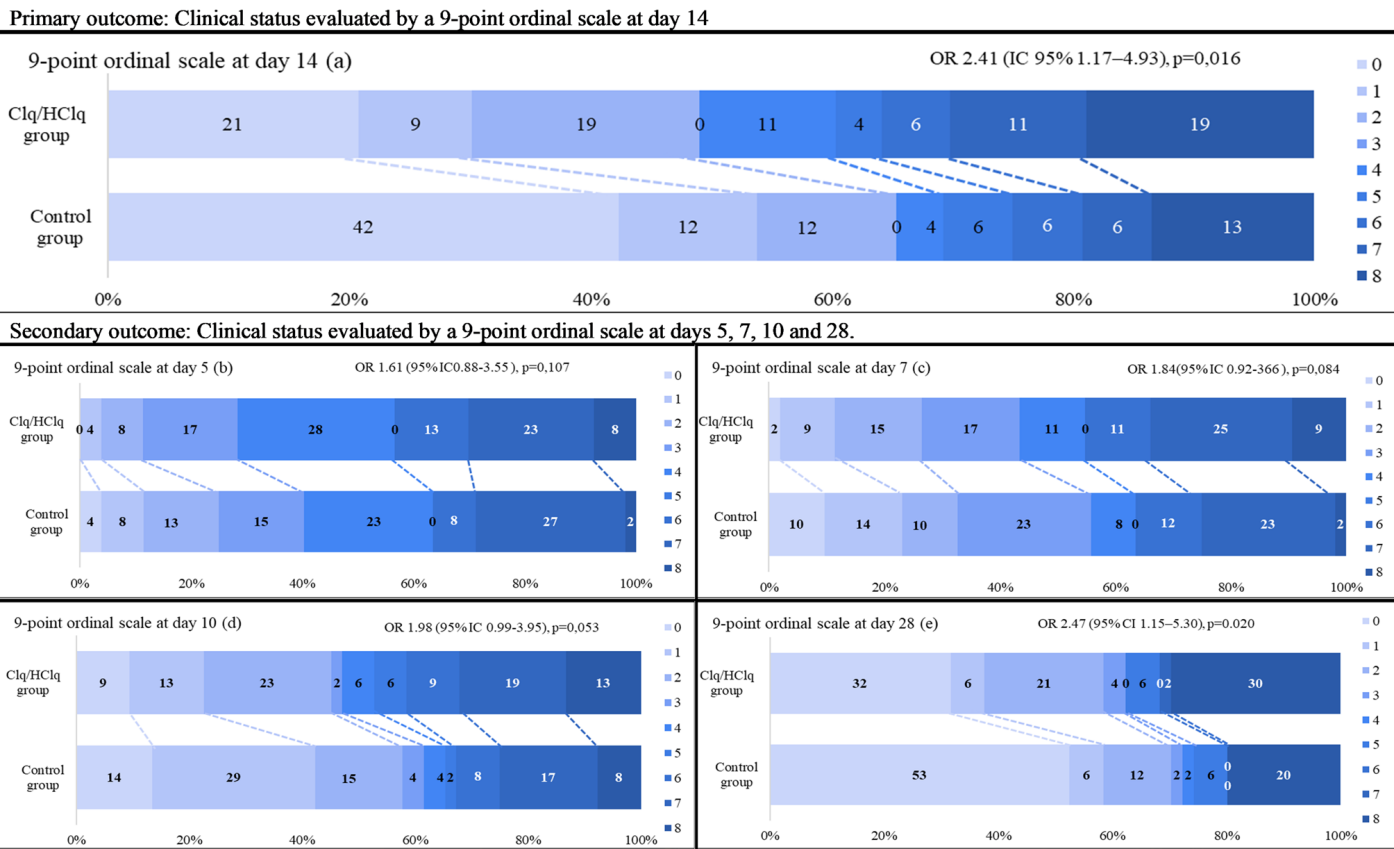
Primary and secondary outcomes in the mITT population from presentation to the evaluation of clinical status with a 9-point ordinal scale. **(a)** Clinical status evaluated by a 9-point ordinal scale at day 14. **(b)** Clinical status evaluated by a 9-point ordinal scale at day 5. **(c)** Clinical status evaluated by a 9-point ordinal scale at day 7. **(d)** Clinical status evaluated by a 9-point ordinal scale at day 10. **(e)** Clinical status evaluated by a 9-point ordinal scale at day 28; one patient in the control group did not have an ordinal scale score ascertained at day 28 because of loss to follow-up. The scores on the scale were defined as follows: (0) nonhospitalized and no clinical or virological evidence of infection; (1) nonhospitalized and no limitation on activities; (2) nonhospitalized, but with limitation on activities; (3) hospitalized, but not requiring supplemental oxygen; (4) hospitalized and on oxygen via mask or nasal prongs; (5) hospitalized, on noninvasive ventilation or high-flow oxygen or pressure support ventilation in weaning mode; (6) hospitalized, intubated and on MV; (7) hospitalized on MV and additional organ support (renal replacement therapy, vasoactive drugs or extracorporeal membrane oxygenation), and (8) dead. The percentages shown have been rounded to whole numbers. ORs (95% CIs) and *p* values were derived from ordinal logistic regression, assuming proportional ORs, adjusted for age and baseline severity (according to ventilatory support) for the mITT population. An OR > 1.00 represents a clinical worsening assessed with the ordinal scale in the Clq/HClq group compared with the control group.

The primary outcome was ascertained in all patients in the mITT population. On the 14th day after randomization, the proportional odds of being in a worse clinical condition according to the 9-point ordinal scale was higher in the Clq/HClq group than in the control group (OR 2.45 [95% CI 1.17 to 4.93], p = 0,016; Fig. 2a), even after controlling for age and MV use at randomization (Supplementary Table S2).

On the 28th day, the proportional odds of being in a worse clinical condition according to the 9-point ordinal scale in the Clq/HClq group was also significant (OR 2.47 [95% CI 1.15–5.30], p = 0.020; Fig. 2e). There were no differences between groups on the 5th day (OR 1.61 (95% CI 0.88–3.55), p = 0.107; Fig. 2b), 7th day (OR 1.84 (95% CI 0.2–3.66), p = 0.084; Fig. 2c) or 10th day (OR 1.98 (95% CI 0.99–3.95), p = 0.053; Fig. 2d) (Supplementary Table S2).

**Figure 3:**
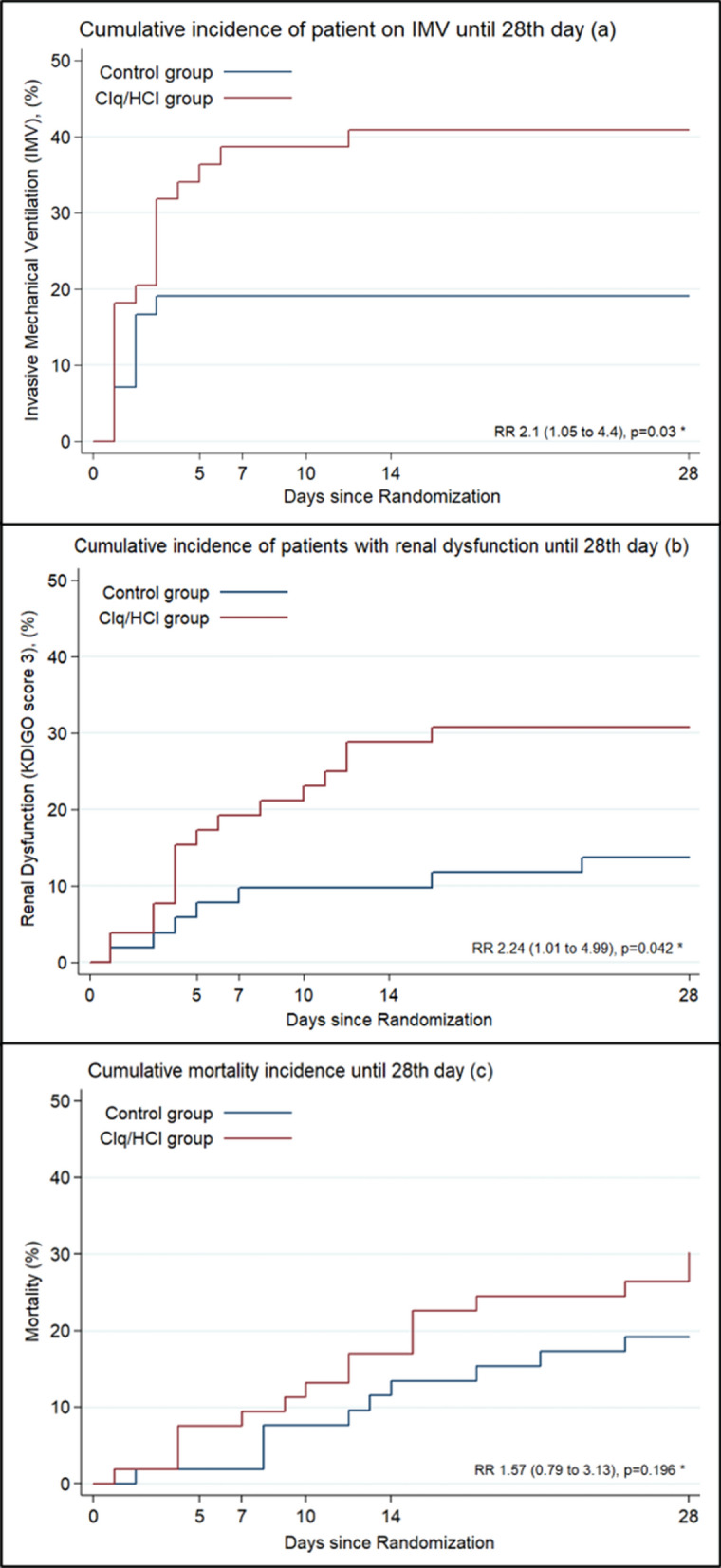
Cumulative incidence of IMV, acute renal dysfunction and mortality until the 28th day. **(a)** Considering the 44 patients from the Clq/HClq group and 42 from the control group of the mITT population who were not on MV at baseline. **(b)** Considering the 52 patients from the Clq/HClq group and 51 from the control group of the mITT population who were not at KDIGO stage 3 at baseline. **(c)** Considering the entire mITT population. *RRs with CIs calculated using the Wald likelihood test.

The cumulative incidence of nonintubated enrolled patients progressing to needing MV was higher in the Clq/HClq group than in the control group (18 [40.9%] vs 8 [19,9%]; RR 2.15 [95% CI 1.05 to 4.4], p = 0.030; Fig. 3a), as was the cumulative incidence of acute renal dysfunction at any point from randomization until the 28th day (17 [32.1%] vs 8 [15.4%]; RR 2.24 [95% CI 1.01–4.99], p = 0.048; Fig. 3b).

The 28-day mortality was not different between the two groups (RR 1.57 [95% CI 0.79 to 3.13], p = 0.196; Fig. 3c). There was no significant difference between groups regarding the number of ventilator-free days, death, the length of ICU stay or the length of hospital stay among survivors until the 28th day after randomization (Table 2). SOFA scores, CRP levels, neutrophil/lymphocyte ratios on days 5, 7, 10, 14 and 28 (Supplementary Table S3) and the development of coagulopathy until the 28th day (Table 2) were not different between groups.

**Table 2:** Other secondary and safety outcomes.

Secondary outcomes	Clq/HClq group	Control group	Difference (95% CI)	p value
**Secondary outcomes until 28th day of the study period**	**(n = 53)**	**(n = 52)**		
MV-free days, median (IQR), days	25 (3–28)	28 (4–28)	− 3 (− 11.7 to 5.7)^a^	0.236
ICU LOS among survivors, median (IQR), days^c^	3.5 (1–12)	3 (0–7)	1 (− 2.6 to 4.6)^a^	0.368
Hospital LOS among survivors, median (IQR), days^c^	7.5 (5–16)	7 (4–12)	1 (− 2.9 to 4.9)^a^	0.257
Coagulopathy incidence, no (%)^d^	28 (62.2)	26 (61.9)	1.01 (0.72 to 1.39)^b^	0.976
IMV incidence, no (%)^e^	18 (41)	8 (19)	2.15 (1.05 to 4.40)^b^	0.030
Acute renal dysfunction incidence, no (%)^f^	16 (31)	7 (14)	2.24 (1.01 to 4.99)^b^	0.042
Mortality, no (%)	16 (30)	10 (19)	1.57 (0.79 to 3.13)^b^	0.196
**Safety outcome**^**g**^	**(n = 68)**	**(n = 70)**		
Arrhythmias, no (%)	4 (5.9)	1 (1.9)	3.92 (0.45 to 33.9)^b^	0.176

*KDIGO* Kidney Disease: Improving Global Outcomes; *IMV* invasive mechanical ventilation; *ICU* intensive care unit; *LOS* length of stay; *HFNC* high-flow nasal cannula; *NIPPV* noninvasive positive-pressure ventilation; *IQR* interquartile range; *Clq* chloroquine; *HClq* hydroxychloroquine; *CI* confidence interval.

^a^Median difference with corresponding 95% CI calculated as an asymmetric Laplace distribution.

^b^Risk ratios (RRs) with CIs calculated using the Wald likelihood test.

^c^Considering the 38 survivors from the Clq/HClq group and 42 from the control group of the modified intention to treat (mITT) population.

^d^Considering 45 from the Clq/HClq group and 43 from the control group of the mITT population who had no coagulopathy at baseline.

^e^Considering the 44 patients from the Clq/HClq group and 42 from the control group of the mITT population who were not on MV at baseline.

^f^Considering the 52 patients from the Clq/HClq group and 51 from the control group of the mITT population who were not at KDIGO stage 3 at baseline.

^g^Safety population consisted of 137 patients, of whom 67 were in the Clq/HClq group and 70 were in the control group.

The safety population consisted of 138 patients, of whom 68 were in the Clq/HClq group and 70 were in the control group. There was no difference between groups regarding the proportion of patients with arrhythmia (4 [5.9%] in the Clq/HClq group vs 1 [1.4%] in the control group (RR 3.92 [95% CI 0.45 to 33.95]), p = 0.176; Table 2). Among patients who had arrhythmia, only one patient in the Clq/HClq group stopped treatment on the third day owing to severe arrhythmia as an adverse event. There were no adjudicated deaths due to arrhythmia.

In comparisons of the Clq, HClq and control groups, no significant difference in the primary, secondary or safety outcomes was found between the Clq and HClq subgroups (Supplementary Tables S4 and S5). This finding supports the results of the comparison between the intervention group (composed of patients who received Clq or HClq) and the control group.

## Discussion

In this trial, Clq/HClq in the prescribed doses was inferior to standard care alone, reflected by the worsening clinical status of patients with severe COVID-19 evaluated on the 14th and 28th days after randomization (Fig. 2A,E). Moreover, patients who received Clq/HClq showed higher cumulative incidences of IMV and renal dysfunction until the 28th day (Fig. 3A,B). Despite this, patients in the two groups had a similar number of ventilator-free days and lengths of ICU and hospital stay (Table 2). Mortality was also numerically higher in the Clq/HClq group, but the difference was not statistically significant (Fig. 3C). Other secondary outcomes, such as SOFA scores, CRP levels and neutrophil/lymphocyte ratios at admission and the evaluated hospitalization days, showed no difference^21^. The presence of coagulopathy until the 28th day was similar in both groups.

Our trial enrolled severe COVID-19 patients who were receiving supplemental oxygen or MV, and most of them were admitted to the ICU. Clq/HClq was started relatively early after symptom onset (median, 7 days), which is earlier than the median time from symptom onset to intervention start in a trial of remdesivir treatment for COVID-19 but similar to the start time in the Coalition Covid-19 Brazil I Trial^8,22^.

The use of Clq/HClq in most nonrandomized studies and in randomized clinical trials so far, especially in mild or moderate COVID-19 patients, has not shown any associated benefit in terms of mortality or clinical improvement^23–26^. In the Coalition Covid-19 Brazil I trial, HClq with or without azithromycin, when compared to standard care, did not result in clinical improvement or harm in patients admitted to the hospital with mild to moderate COVID-19^8^. Our trial selected patients with more severe forms of COVID-19 pneumonia, which may account, at least in part, for the differences we found.

Despite this controversy, in Brazil, Clq/HClq has been formally recommended for the treatment of COVID-19 by the Ministry of Health for severe cases since March 27, 2020, and for mild cases since May 21, 2020^14,15^. Therefore, we opted to test Clq/HClq at a dose that was lower than that in other trials but officially suggested in Brazil and in many international protocols at the beginning of the trial^8,9,14–16,23,27^. Even with a low dose of Clq/HClq in only severe COVID-19 patients, we found a clearly harmful clinical outcome on the 14th and 28th days and increased accumulated incidences in the need for IMV and renal dysfunction until the 28th day.

Our results showed that the intervention increased the risk of IMV in patients who were admitted with hypoxemia (SaO_2_ < 94%) but not yet intubated. Not totally surprisingly, since the intervention medications tested have not been shown to inhibit the infection of human lung cells with SARS-CoV-2^28^, the RECOVERY trial has also revealed a tendency for the same result in similar patients receiving a higher dose of HClq^9^.

The increased incidence of renal dysfunction with Clq/HClq can be explained by the potential renal toxicity of the drugs and their possible interaction with SARS-CoV-2 in the kidney^29^. Clq and HClq have a large distribution volume. Clq has an approximate half-life of 50 days and a high renal clearance, while HClq has a 30-day half-life and takes approximately 6 months to be fully eliminated^30^. After the initial proliferation, SARS-CoV-2 disseminates through the circulation and can persist in the kidney, contributing to the high rate of renal dysfunction in more severe forms of COVID-19. Thus, the association of these drugs and the virus in the kidney can be devastating and could explain the increase in the incidence of renal dysfunction in the Clq/HClq group. One of the main safety concerns with Clq/HClq treatment is related to cardiovascular effects, especially arrhythmias related to QT interval prolongation^17^. In this study, there was a small incidence of arrhythmias and no significant differences between groups. The discrepancy in the results might be related to the fact that we excluded patients with any kind of cardiopathy or arrhythmia at baseline and used the lowest proposed dose^17^. Therefore, we have shown that Clq or HClq, in the doses used here, are deleterious to patients with severe forms of COVID-19.

The WHO 9-point ordinal scale is the most widely used scale to measure clinical progression outcome^12^ in phase III RCTs involving patients with COVID-19, as in our study^31,32^. According to Desai and Gyawali^31^, 43% of clinical trials for the treatment of COVID-19 in 2020 adopted this scale to evaluate outcomes. This instrument has been considered the best way to measure respiratory support over 14 days because it captures the entire spectrum of the clinical disease, from no symptoms to death, and provides greater resolution at the most severe end of the disease spectrum^32^. Although the scale is objective, the decision to perform tracheal intubation and its timing depends on the operator, which may be a limitation of an open and pragmatic trial tested in the real world. We do not believe these characteristics interfered with the results, since the important prognostic variables were well balanced between the two groups and outcomes were based on hard data.

In conclusion, the addition of Clq/HClq to standard care in patients admitted to the hospital with severe COVID-19 resulted in clinical worsening and higher incidences of IMV and renal dysfunction, even though there was no difference in mortality. According to these findings, the use of Clq/HClq in patients with more severe forms of COVID-19 pneumonia is strongly contraindicated, and these results can inform clinical practice and guidelines.

## Supplementary Information

Supplementary Information.

## Data Availability

The datasets used and/or analyzed during the current study are available from the corresponding author on reasonable request.
