# Supplementary material for: An open-label randomized controlled trial evaluating the efficacy of chloroquine/hydroxychloroquine in severe COVID-19 patients
Source: Sci Rep. 2021 Apr 27;11:9023. doi: 10.1038/s41598-021-88509-9 (PMC8079411; doi:10.1038/s41598-021-88509-9)
Supplement: Supplementary file 1 — Supplementary Information. [file 41598_2021_88509_MOESM1_ESM.pdf]

SUPPLEMENTARY MATERIAL

An open-label randomized controlled trial evaluating the efficacy of chloroquine/hydroxychloroquine in severe COVID-19 patients

Álvaro Réa-Neto [1, 2], Rafaella Stradiotto Bernardelli [2], Bruna Martins Dzivielevski Câmara [2], Fernanda Baeumle Reese [2], Marcos Vinicius Oliveira Queiroga [2], Mirella Cristine Oliveira [2]

Authors Affiliations

[1] Department of Medicine, Federal University of Paraná (UFPR), Curitiba, Brazil

[2] CEPETI, Center for Study and Research in Intensive Care Medicine, Curitiba, Brazil

SUPPLEMENTARY MATERIAL CONTENTS

Additional Statistical Analysis………………………………………………………….. 3

Supplementary Table 1: Chloroquine (Clq), Hydroxychloroquine (HClq) and control groups baseline characteristics and concomitant treatments during hospitalization, considering the modified intention to treat population………..……..…………………. 4

Supplementary Table 2: Primary and secondary outcomes presentation to clinical status evaluated according to a 9-point ordinal scale at modified intention to treat population, comparison between Clq/HClq and control groups……………….….….……………... 6

Supplementary Table 3: Comparison of SOFA scores; CRP and Neutrophils/Lymphocytes ratio between Clq/HClq and control groups during hospitalization on days 5-7-10-14 and 28 after randomization at modified intention to treat population ..……………………...………………….……...………………………… 8

Supplementary Table 4: Comparison among Chloroquine (Clq), Hydroxychloroquine (HClq) and control groups primary and secondary outcomes presentation to clinical status evaluated according to a 9-point ordinal scale at modified intention to treat population.. 9

Supplementary Table 5: Comparison among Chloroquine (Clq), Hydroxychloroquine (HClq) and control groups others secondary and safety outcomes at modified intention to treat population ……...…...……………………….……………………………….………….. 11

Additional Statistical Analysis

Additional statistical analysis was performed to describe and compare the outcomes among chloroquine (Clq); hydroxychloroquine (HClq); and control groups, since the intervention group was composed by patients receiving chloroquine or hydroxychloroquine.

In the primary outcome analysis, we assessed the effect of the Clq group and the HClq group in relation to the control group, in addition to the Clq group comparing to the HClq group, on the 9-point ordinal scale on day 14, as an OR and confidence interval (CI) derived from an ordinal logistic regression, assuming proportional ORs adjusted for age and severity at the baseline (under invasive mechanical ventilation or not at randomization). The same regression model was used to analyze the secondaries outcomes on same scale on days 5, 7, 10 and 28 after randomization.

The effect of the Clq group and the HClq group in relation to the control group, in addition to the Clq group comparing to the HClq group on the mortality, the incidences of invasive mechanical ventilation, acute renal dysfunction by KDIGO stage 3 and coagulopathy in any moment until the 28th day after randomization are reported as proportions and differences between groups as risk ratios (RRs) with CIs, calculated by using the Wald likelihood test.

The effect of the Clq group and the HClq group in relation to the control group, in addition to the Clq group comparing to the HClq group in the number of MV-free days, the length of stay in the ICU and the length of stay in the hospital for 28 days was compared by median differences calculated with a quantile regression based on an asymmetric Laplace distribution.

A *p* value less than 0.05 was considered statistically significant in all analyses. There was no missing value for the primary outcome, so imputation was not necessary. The analyses were performed through the Stata software, version 17.

Supplementary Table 1: Chloroquine (Clq), Hydroxychloroquine (HClq) and control groups baseline characteristics and concomitant treatments during hospitalization, considering the modified intention to treat population.

**Characteristics**	**Clq group (n=24)**	**HClq group (n=29)**	**Control group (n=52)**
Age, mean (SD), years	55 (10.4)	54.5 (13.6)	52.8 (12.6)
Sex Male, No. (%)	17 (70.8)	19 (65.5)	34 (65.4)
Body mass index, mean (SD)	31.4 (5.7)	31.2 (5.9)	30.8 (5.5)
**Comorbidities,** No. (%) [a]			
Hypertension	11 (45.8)	8 (27.6)	21 (40.4)
Diabetes Mellitus	7 (29.2)	4 (13.8)	16 (30.8)
Chronic lung disease[b]	2 (8.4)	4 (13.7)	3 (5.8)
Immunocompromised state[c]	1 (4.2)	1 (3.4)	4 (7,6)
Charson Score, median (IQR)	0.6 (0-1)	0.5 (0-1)	0.7 (0-1)
SpO_2 < 94%, No. (%)	24 (100)	27 (93.1)	49 (94.2)
MV at baseline, No. (%)	7 (29.2)	2 (6.9)	10 (19.2)
In ICU at baseline, No. (%)	24 (100)	20 (69)	41 (78.8)
Pulmonary ground-glass opacities at CT scan, No. (%)	24 (100)	28 (96.6)	49 (94.2)
Time from symptom onset to randomization, median (IQR), days	8 (5 - 10)	8 (6 - 10)	7 (5 - 10)
APACHE II Score, median (IQR)	8 (5.25 - 13)	10 (5.5 - 12)	8 (5 - 12.8)
SOFA Score, median (IQR)	4 (2 - 5)	2 (1 – 3)	2.5 (1 - 4)
KDIGO, No. (%)			
0	18 (75)	26 (89.7)	40 (76.9)
1	5 (20.8)	2 (6.9)	9 (17.3)
2	1 (4.2)	0 (0)	2 (3.8)
3	0 (0)	1 (3.4)	1 (1.9)
Coagulopathy at baseline, No. (%)	5 (20.8)	3 (10.3)	9 (17.3)
**Score on 9-point ordinal scale, No. (%)[d]**			
3: hospitalized, but not requiring supplemental oxygen	0 (0)	1 (1.9)	0 (0)
4: hospitalized and on oxygen by mask or nasal prongs	17 (70.8)	26 (89.7)	42 (80.8)
5: hospitalized, on NIPPV, HFNC or support pressure MV in weaning mode	0 (0)	0 (0)	0 (0)
6: hospitalized, intubated and on MV	1 (4.2)	0 (0)	3 (5.8)
7: hospitalized on MV and additional organ support (hemodialysis and/or vasoactive drugs and/or ECMO)	6 (25)	2 (6.9)	7 (13.5)
**Laboratory variables**			
Hemoglobin, mean (SD), g/dL[e]	13.2 (1.9)	13.7 (1.3)	13.9 (1.3)
White blood cell, median (IQR), $x10^9/L$[e]	7.9 (6.1 - 12.6)	7.8 (6.1 - 10.8)	7.4 (5.2 - 9)
Lymphocyte count, median (IQR), $x10^9/L$[e]	1.17 (0.64 - 1.53)	0.83 (0.72 - 1.28)	0.87 (0.56 - 0.90)
Neutrophil/Lymphocyte ratio, median (IQR)[e]	5.8 (3.8 - 12.9)	6.4 (4.1 - 9.5)	6.5 (4.0 - 9.7)
Platelet count, mean (SD), $x10^9/L$[e]	215.8 (78.8)	229.3 (80.2)	217.2 (78.0)
Serum creatinine, median (IQR), mg/dL	0.83 (0.64 - 1.07)	0.76 (0.66 - 1.01)	0.78 (0.63 - 1.00)
CRP, median (IQR), mg/L[f]	89.5 (65 - 152)	103.8 (75.8 - 174.1)	84 (41.5 - 151.0)

D-dimer, median (IQR), nmol/L[g]	1011.5 (455 - 2023)	637.7 (222.9 - 1106)	821 (409 -1601.3)
**Concomitant medications**			
Corticosteroids, No. (%)	13 (54.2)	24 (82.8)	39 (75)
Oseltamivir, No. (%)	17 (70.8)	10 (34.5)	27 (51.9)
Azithromycin, No. (%)	24 (100)	27 (93.1)	43 (82.7)

Abbreviations: SpO2: pulse oxygen saturation; ICU: intensive care unit; MV: mechanical ventilation; IMV: invasive ventilation; CT: computed tomography; APACHE II: Acute Physiology and Chronic Health disease Classification System II; SOFA: Sequential Organ Failure Assessment; KDIGO: Kidney Disease: Improving Global Outcomes; HFNC: high-flow nasal cannula; NIPPV: noninvasive positive-pressure ventilation; ECMO: extracorporeal membrane oxygenation; CRP: C-reactive protein. SD, standard deviation; IQR, interquartile range; Clq, chloroquine; HClq, hydroxychloroquine.

a No participants had any of the following comorbidities: chronic renal failure; peripheral vascular insufficiency; or heart, liver, rheumatic, or hematological disease.

b Considering asthma or chronic obstructive pulmonary disease.

c Considering cancer or human immunodeficiency virus infection.

d Only hospitalized patients were eligible for the trial; therefore, patients who had scores of 0, 1, 2 or 8 on a nine-point ordinal scale were not eligible.

[e] One missing data in the HClq group.

[f] Two missing data in the Clq group, one missing data in the HClq group and one at the control group.

[g] Sex missing data in the Clq group, 21 missing data in the HClq group and 25 in the control group.

Supplementary Table 2: Primary and secondary outcomes presentation to clinical status evaluated according to a 9-point ordinal scale at modified intention to treat population, comparison between Clq/HClq and control groups

Outcome	Clq/HClq group (n=53)	Control group (n=52)	OR (95% CI)	*p* value
**Primary outcome:**				
**9-point ordinal score on day 14**			2.41 (1.17 to 4.93)[a]	0.016
0: nonhospitalized and no clinical or virological evidence of infection	11 (20.8)	22 (42.3)		
1: nonhospitalized and no limitation on activities	5 (9.4)	6 (11,5)		
2: nonhospitalized, but with limitation on activities	10 (18.9)	6 (11.5)		
3: hospitalized, but not requiring supplemental oxygen	0 (0)	0 (0)		
4: hospitalized and on oxygen via mask or nasal prongs	6 (11.3)	2 (3.8)		
5: hospitalized, on NIPPV, HFNC or support pressure MV in weaning mode	2 (3.8)	3 (5.8)		
6: hospitalized, intubated and on MV;	3 (5.7)	3 (5.8)		
7: hospitalized on MV and additional organ support (renal replacement therapy, vasoactive drugs or extracorporeal membrane oxygenation)	6 (11.3)	3 (5.8)		
8: dead.	10 (18.9)	7 (13.5)		
**Secondary outcomes:**				
**9-point ordinal score on day 5**			1.61 (0.88 to 3.55)[a]	0.107
0: nonhospitalized and no clinical or virological evidence of infection	0 (0)	2 (3,8)		
1: nonhospitalized and no limitation on activities	2 (3.8)	4 (7.7)		
2: nonhospitalized, but with limitation on activities	4 (7.5)	7 (13.5)		
3: hospitalized, but not requiring supplemental oxygen	9 (17)	8 (15.4)		
4: hospitalized and on oxygen via mask or nasal prongs	15 (28.3)	12 (23.1)		
5: hospitalized, on NIPPV, HFNC or support pressure MV in weaning mode	0 (0)	0 (0)		
6: hospitalized, intubated and on MV;	7 (13.2)	4 (7.7)		
7: hospitalized on MV and additional organ support (renal replacement therapy, vasoactive drugs or extracorporeal membrane oxygenation)	12 (22.6)	14 (26.9)		
8: dead.	4 (7.5)	1 (1.9)		
**9-point ordinal score on day 7**			1.84 (0.92 to 3.66)[a]	0.107
0: nonhospitalized and no clinical or virological evidence of infection	1 (1.9)	5 (9.6)		
1: nonhospitalized and no limitation on activities	5 (9.4)	7 (13.5)		
2: nonhospitalized, but with limitation on activities	8 (15.1)	5 (9.6)		
3: hospitalized, but not requiring supplemental oxygen	9 (17)	12 (23.1)		

4: hospitalized and on oxygen via mask or nasal prongs	6 (11.3)	4 (7.7)		
5: hospitalized, on NIPPV, HFNC or support pressure MV in weaning mode	0 (0)	0 (0)		
6: hospitalized, intubated and on MV	6 (11.3)	6 (11.5)		
7: hospitalized on MV and additional organ support (renal replacement therapy, vasoactive drugs or extracorporeal membrane oxygenation)	13 (24.5)	12 (23.1)		
8: dead.	5 (9.4)	1 (1.9)		
**9-point ordinal score on day 10**			1.98 (0.99 to 3.95)[a]	0.053
0: nonhospitalized and no clinical or virological evidence of infection	5 (9.4)	7 (13.5)		
1: nonhospitalized and no limitation on activities	7 (13.2)	15 (28.8)		
2: nonhospitalized, but with limitation on activities	12 (22,6)	8 (15,4)		
3: hospitalized, but not requiring supplemental oxygen	1 (1,9)	2 (3,8)		
4: hospitalized and on oxygen via mask or nasal prongs	3 (5.7)	2 (3.8)		
5: hospitalized, on NIPPV, HFNC or support pressure MV in weaning mode	3 (5.7)	1 (1.9)		
6: hospitalized, intubated and on MV	5 (9.4)	4 (7.7)		
7: hospitalized on MV and additional organ support (renal replacement therapy, vasoactive drugs or extracorporeal membrane oxygenation)	10 (18.9)	9 (17.3)		
8: dead.	7 (13.2)	4 (7.7)		
**9-point ordinal score at day 28**			2.47 (1.15-5.30)[a]	0.020
0: nonhospitalized and no clinical or virological evidence of infection	17 (32.1)	27 (52.9)		
1: nonhospitalized and no limitation on activities	3 (5.7)	3 (5.9)		
2: nonhospitalized, but with limitation on activities	11 (20.8)	6 (11.8)		
3: hospitalized, but not requiring supplemental oxygen	2 (3,8)	1 (2)		
4: hospitalized and on oxygen via mask or nasal prongs	0 (0)	1 (2)		
5: hospitalized, on NIPPV, HFNC or support pressure MV in weaning mode	3 (5.7)	3 (5.9)		
6: hospitalized, intubated and on MV	0 (0)	0 (0)		
7: hospitalized on MV and additional organ support (renal replacement therapy, vasoactive drugs or extracorporeal membrane oxygenation)	1 (1.9)	0 (0)		
8: dead.	16 (30.2)	10 (19.6)		

Abbreviations: MV: mechanical ventilation; ICU, intensive care unit; LOS, length of stay; HFNC, high-flow nasal cannula; NIPPV, noninvasive positive-pressure ventilation; Clq, chloroquine; HClq, hydroxychloroquine.

Data are n (%). One patient in the control group did not have ordinal scale status ascertained in 28 days for missing the follow-up; however, the clinical status was ascertained in 5, 7, 10 and 14 days.

[a] Odds Ratio (OR) and confidence interval (CI) and *p* value derived from an ordinal logistic regression, assuming proportional ORs adjusted for age and severity at the baseline (under MV or not at randomization) for the modified intention to treat (mITT) population. OR >1·00 represents a clinical worsening assessed on the ordinal scale in the Clq/HClq group compared with the control group.

Supplementary Table 3: Comparison of SOFA scores; CRP and Neutrophil/Lymphocyte ratio between Clq/HClq and control groups during hospitalization on days 5-7-10-14 and 28 after randomization at modified intention to treat population.

Secondary Outcomes	Clq/HClq group (n=53)	Control group (n=52)	Difference (95% CI)[f]	*p* value[g]
**SOFA Score**				
**D5[a]**	3.5 (2 to 6)	4 (1 to 7)	0 (-2.49 to 2.49)	0.972
**D7[b]**	4 (1 to 8)	4.5 (2 to 7)	-1 (-4.21 to 2.21)	0.741
**D10[c]**	5 (3 to 7)	6 (4 to 7)	-1 (-3.44 to 1.44)	0.493
**D14[d]**	5 (3 to 8)	4.5 (3 to 8.5)	0 (-3.66 to 3.6)	0.915
**D28[e]**	5 (5 to 8)	6.5 (6 to 7)	-1 (-4.21 to 2.21)	0.620
**CRP, mg/L**				
**D5[a]**	113.3 (28 to 165.5)	131.5 (27.8 to 188.2)	-7.3 (-83.7 to 69.1)	0.633
**D7[b]**	125.1 (77.3 to 269)	103 (19.1 to 183.7)	14 (-84.6 to 112.6)	0.140
**D10[c]**	129 (50.2 to 229)	138.5 (92.1 to 183.7)	-9.5 (-99.6 to 80.6)	0.760
**D14[d]**	134.6 (38.4 to 258)	178.5 (41.7 to 262.1)	30.3 (-113.5 to 174.1)	0.767
**D28[e]**	162.6 (92 to 170)	99 (72.1 to 128.3)	54.4 (-452.4 to 161.2)	0.221
**Neutrophil/ Lymphocyte ratio**				
**D5[a]**	6.6 (5.1 to 8.5)	5.1 (3.3 to 9.3)	1.6 (-0.6 to 3.8)	0.389
**D7[b]**	8.5 (5.6 to 11.1)	7.6 (5.1 to 10.8)	0.8 (-2.5 to 4.1)	0.774
**D10[c]**	9.5 (6.5 to 14.2)	7.6 (5.2 to 11)	1.9 (-2.9 to 6.7)	0.352
**D14[d]**	7.3 (5.3 to 9.7)	10.1 (4.5 to 24.2)	-7.3 (-15.7 to 1.1)	0.783
**D28[e]**	6.3 (5.3 to 8.3)	5.8 (5.1 to 10.6)	0,33 (-5.7 to 6.4)	1

Data are median (interquartile range). SOFA: Sequential Organ Failure Assessment; CRP: C-reactive protein.
[a] On D5, 38 patients in the Clq/HClq group and 33 in the control group who were still hospitalized were considered for this mITT analysis.
[b] On D7, 31 patients in the Clq/HClq group and 26 in the control group who were still hospitalized were considered for this mITT analysis.
[c] On D10, 22 patients in the Clq/HClq group and 15 in the control group who were still hospitalized were considered for this mITT analysis.
[d] On D14, 18 patients in the Clq/HClq group and 12 in the control group who were still hospitalized were considered for this mITT analysis.
[e] On D28, 5 patients in the Clq/HClq group and 4 in the control group who were still hospitalized were considered for this mITT analysis.
[f] Median difference with corresponding 95% CI calculated as an asymmetric Laplace distribution.
[g] Wilcoxon rank-sum test significance.

Supplementary Table 4: Comparison among Chloroquine (Clq), Hydroxychloroquine (HClq) and control groups primary and secondary outcomes presentation to clinical status evaluated according to a 9-point ordinal scale at Modified Intention-to-Treat Population.

Outcome	Clq group (n=24)	HClq group (n=29)	Control group (n=52)	Clq vs. Control	HClq vs. Control	Clq vs. HClq
**Primary outcome:**						
**9-point ordinal score on day 14**				2.64 (1.11 to 6.31) 0.029 [a]	2.19 (0.92 to 5.21) 0.075 [a]	1.20 (0.45 to 3.20) 0.710 [a]
0	2 (8.3)	9 (31)	22 (42.3)			
1	2 (8.3)	3 (10.3)	6 (11,5)			
2	6 (25.0)	4 (13.8)	6 (11.5)			
3	0 (0)	0 (0)	0 (0)			
4	4 (16.7)	2 (6.9)	2 (3.8)			
5	1 (4.2)	1 (3.4)	3 (5.8)			
6	2 (8.3)	1 (3.4)	3 (5.8)			
7	3 (12.5)	3 (10.3)	3 (5.8)			
8	4 (16.7)	6 (20.7)	7 (13.5)			
**Secondary outcomes:**						
**9-point ordinal score on day 5**				1.71 (0.71 to 4.09) 0.230 [a]	1.83 (0.79 to 4.22) 0.157 [a]	0.93 (0.35 to 2.52) 0.892 [a]
0	0 (0)	0 (0)	2 (3,8)			
1	0 (0)	2 (6.9)	4 (7.7)			
2	2 (8.3)	2 (6.9)	7 (13.5)			
3	4 (16.7)	5 (17.2)	8 (15.4)			
4	4 (16.7)	11 (37.9)	12 (23.1)			
5	0 (0)	0 (0)	0 (0)			
6	6 (25.0)	1 (3.4)	4 (7.7)			
7	7 (29.2)	5 (17.2)	14 (26.9)			
8	1 (4.2)	3 (10.3)	1 (1.9)			
**9-point ordinal score on day 7**				1.79 (0.76 to 4.22) 0.180 [a]	1.87 (0.81 to 4.35) 0.143 [a]	0.96 (0.35 to 2.58) 0.931 [a]
0	0 (0)	1 (3.4)	5 (9.6)			
1	0 (0)	5 (17.2)	7 (13.5)			
2	6 (25.0)	2 (6.9)	5 (9.6)			
3	2 (8.3)	7 (24.1)	12 (23.1)			
4	2 (8.3)	4 (13.8)	4 (7.7)			
5	0 (0)	0 (0)	0 (0)			
6	5 (20.8)	1 (3.4)	6 (11.5)			
7	8 (33.3)	5 (17.2)	12 (23.1)			
8	1 (4.2)	4 (13.8)	1 (1.9)			
**9-point ordinal score on day 10**				2.20 (0.94 to 5.14) 0.068 [a]	1.53 (0.66 to 3.55) 0.323 [a]	1.44 (0.54 to 3.85) 0.467 [a]
0	0 (0)	5 (17.2)	7 (13.5)			
1	3 (12.5)	6 (20.7)	15 (28.8)			
2	6 (25.0)	4 (13.8)	8 (15,4)			
3	1 (4.2)	0 (0)	2 (3,8)			
4	0 (0)	3 (10.3)	2 (3.8)			
5	1 (4.2)	2 (6.9)	1 (1.9)			
6	4 (16.7)	1 (3.4)	4 (7.7)			
7	7 (29.2)	3 (10.3)	9 (17.3)			
8	2 (8.3)	5 (17.2)	4 (7.7)			

**9-point ordinal score at day 28**				2.75 (1.10 to 6.88) 0.031 [a]	2.23 (0.89 to 5.58) 0.088 [a]	1.23 (0.33 to 3.44) 0.689 [a]
0	4 (16.7)	13 (44.8)	27 (52.9)			
1	2 (8.3)	1 (3.4)	3 (5.9)			
2	7 (29.2)	4 (13.8)	6 (11.8)			
3	2 (8.3)	0 (0)	1 (2)			
4	0 (0)	0 (0)	1 (2)			
5	1 (4.2)	2 (6.9)	3 (5.9)			
6	0 (0)	0 (0)	0 (0)			
7	1 (4.2)	0 (0)	0 (0)			
8	7 (29.2)	9 (31.0)	10 (19.6)			

Data are n (%). One patient in the control group did not have ordinal scale status ascertained in 28 days for missing the follow-up; however, the clinical status was ascertained in 5, 7, 10 and 14 days.

The scores on the scale were defined as follows: (0) nonhospitalized and no clinical or virological evidence of infection; (1) nonhospitalized and no limitation on activities; (2) nonhospitalized, but with limitation on activities; (3) hospitalized, but not requiring supplemental oxygen; (4) hospitalized and on oxygen via mask or nasal prongs; (5) hospitalized, on noninvasive ventilation or high-flow oxygen or pressure support ventilation in weaning mode; (6) hospitalized, intubated and on MV; (7) hospitalized on MV and additional organ support (renal replacement therapy, vasoactive drugs or extracorporeal membrane oxygenation), and (8) dead.

[a] Odds Ratio (OR) and confidence interval (CI) and *p* value derived from an ordinal logistic regression, assuming proportional ORs adjusted for age and severity at the baseline (under MV or not at randomization) for the modified intention to treat (mITT) population. OR >1·00 represents a clinical worsening assessed on the ordinal scale in the Clq group compared with the control group; HClq group compared with the control group; and Clq group compared with the HClq group.

Supplementary Table 5: Comparison among Chloroquine (Clq), Hydroxychloroquine (HClq) and control groups others secondary and safety outcomes at Modified Intention-to-Treat Population

Secondary outcomes until 28th day study period	Clq group (n=24)	HClq group (n=29)	Control group (n=52)	Clq vs. Control	HClq vs. Control	Clq vs. HClq
MV free days, median (IQR), days	15 (2 – 28)	28 (5 – 28)	28 (4 - 28)	4 (0.36 to 7.64) 0.032[a]	-1 (-4.44 to 2.44) 0.564 [a]	-11 (-23.28 to 1.29) 0.079 [a]
ICU LOS among survivors, median (IQR), days[c]	7 (3 – 14)	2 (0 – 6)	3 (0 - 7)	3 (-1.74 to 7.74) 0.211 [a*]	0 (- 4.38 to 4.38) 1[a*]	3 (-2.56 to 8.56) 0.286 [a*]
Hospital LOS among survivors, median (IQR), days[c]	9 (6- 26)	7 (5-12)	7 (4 - 12)	2 (-3.22 to 7.22) 0.448 [a]	0 (- 4.93 to 4.93) 1[a]	2 (-3.99 to 7.99) 0.508 [a]
Coagulopathy incidence, No (%)[d]	6 (25.0)	4 (13.8)	26 (61.9)	1.44 (1.09 to 1.92) 0.011[b]	1.46 (0.88 to 2.43) 0.142 [b]	2.11(1.31 to 3.39) 0.002 [b]
Invasive mechanical ventilation incidence, No (%)[e]	8 (13.2)	11 (40.7)	8 (19)	2.47(1.11 to 5.1) 0.027[b]	1.94 (0.88 to 4.30) 0.101 [b]	1.27 (0.63 to 2.57) 0.505 [b]
Acute renal disfunction incidence, No (%)[f]	8 (33.3)	8 (28.6)	7 (14)	2.42 (1 to 5.92) 0,047[b]	2.08 (0.84 to 5.14) 0.112 [b]	1.17 (0.52 to 2.63) 0.711 [b]
Mortality, No (%)	7 (29.2)	8 (27.6)	10 (19)	1.52 (0.67 to 3.49) 0.329[b]	1.61 (0.74 to 3.51) 0.228 [b]	0.94 (0.41 to 2.15) 0.883 [b]
**Safety outcome[g]**	**Clq group (n=28)**	**HClq group (n=40)**	**Control group (n=70)**	**Clq vs. Control**	**HClq vs. Control**	**Clq vs. HClq**
Arrhythmias, No (%)	1 (3.6)	3 (7.5)	1 (1.4)	2.5 (0.16 to 38.6) 0.512[b]	5.25 (0.56 to 48.80) 0.145 [b]	0.47 (0.52 to 4.34) 0.511 [b]

Abbreviations: KDIGO: Kidney Disease: Improving Global Outcomes; IMV: invasive mechanical ventilation; ICU, intensive care unit; LOS, length of stay; HFNC, high-flow nasal cannula; NIPPV, noninvasive positive-pressure ventilation; IQR, interquartile range; Clq, chloroquine; HClq, hydroxychloroquine; CI, confidence interval.

[a] Median difference with corresponding 95% CI calculated as an asymmetric Laplace distribution.

[b] Risk ratios (RRs) with CIs calculated using the Wald likelihood test.

[c] Considering the 17 patients fron Clq group, 21 survivors from HClq group and 42 from control group to m-ITT.

[d] Considering the 45 from Clq/HClq group and 43 from control group of m-ITT that were not coagulopathy at baseline.

[e] Considering the 17 patients fron Clq group, 27 patients from HClq group and 42 from control group of m-ITT that were not on MV at baseline.

[f] Considering the 24 patients fron Clq group, 28 patients from HClq group and 51 from control group to mITT that were not KDIGO score 3 at baseline.

[g] Safety population consisted of 138 patients, of whom 28 were in the Clq group, 40 were in the HClq group and 70 were in the control group.

* Adjusted for In ICU at baseline.
